# Body surface mapping of P-waves in sinus rhythm to predict recurrence following cardioversion for atrial fibrillation

**DOI:** 10.3389/fcvm.2024.1417029

**Published:** 2024-07-25

**Authors:** Ibrahim Antoun, Xin Li, Ahmed Kotb, Joseph Barker, Akash Mavilakandy, Ivelin Koev, Zakariyya Vali, Riyaz Somani, G. André Ng

**Affiliations:** ^1^ Department of Cardiology, University Hospitals of Leicester NHS Trust, Glenfield Hospital, Leicester, United Kingdom; ^2^ Department of Engineering, University of Leicester, Glenfield Hospital, Leicester, United Kingdom; ^3^ Department of Cardiovascular Sciences, Clinical Science Wing, University of Leicester, Glenfield Hospital, Leicester, United Kingdom; ^4^ Department of Research, National Institute for Health Research Leicester Research Biomedical Centre, Leicester, United Kingdom

**Keywords:** atrial fibrillation, body surface mapping, p-waves, cardioversion, electrical cardioversion

## Abstract

**Background:**

Direct current cardioversion (DCCV) is used as elective and emergency rhythm control for atrial fibrillation (AF). We aimed to explore the role of P-wave parameters measured during sinus rhythm using body surface mapping (BSM) in predicting successful DCCV for persistent atrial fibrillation (persAF) at 12 months.

**Methods:**

This case–control study included 56 males >18 years old who underwent DCCV for persAF. P-wave parameter collection after DCCV for AF was done using 128 unipolar leads. A band-pass filter of 1–50 Hz was utilised. Corrected P-wave duration (PWDc), P-wave amplitude, and P-wave dispersion were measured to predict 12-month outcomes and time of recurrence.

**Results:**

The mean age was 64 ± 4 years, and 23 patients (44%) were on amiodarone. The 12-month success rate was 44% (*n* = 23), while the rest reverted to AF after 2.6 ± 0.4 months. The parameters were comparable between successful and failed DCCV in the entire cohort and patients not on amiodarone. In patients on amiodarone, patients with failed arms had higher PWDc than those with successful arms (188 vs. 150 ms, *P* = 0.04). Receiver operator characteristic curve analysis for PWDc in the amiodarone cohort showed an area under the curve (AUC) of 0.75 and *P* = 0.049. A recurrence cut-off >161 ms had a sensitivity of 69% and a specificity of 100%, with a hazard ratio of 10.7, *P* = 0.004. The parameters were not predictive of the time of recurrence.

**Conclusion:**

In patients on amiodarone, increased PWDc measured using BSM was associated with higher AF recurrence at 12 months following DCCV for persAF.

## Introduction

Atrial fibrillation (AF) is the most common sustained arrhythmia worldwide, increasing the risk of stroke and mortality (1, 2). AF could be caused by triggers or substrate driven by electrical or structural remodelling. The recent European Society of Cardiology guidelines suggested a rate or rhythm control approach to manage AF (3). Rhythm control options include anti-arrhythmic drugs, catheter ablation, and direct current cardioversion (DCCV). DCCV is particularly useful when the patient is hemodynamically unstable or there is an urgency to resume sinus rhythm (SR). The mechanism of DCCV was proposed to prevent the maintenance of re-entrant tachycardia by the remaining myocardial tissue after depolarisation of a critical mass (4). The 1-year success rate of external DCCV for AF varied between 15% and 47% (5–8). Predicting DCCV outcome after reversion to SR has been explored in the literature. Symptom monitoring was unreliable in predicting DCCV outcomes due to asymptomatic AF (9). Predictors of DCCV outcomes in the literature include demographics, the use of anti-arrhythmic medication (10), cardiac imaging (11), and P-wave analysis (12).

P-wave represents the spatiotemporal convolution of the depolarisation wavefront in the atria as collected in the torso. P-wave parameters provide insights into atrial electrophysiology and essential information regarding atrial remodelling, which triggers and maintains AF (13). These parameters utilised 12-lead ECG and signal-averaged ECG (SAEG) following initial successful cardioversion to predict freedom of arrhythmia rate (Table 1). However, these studies have yet to utilise body surface mapping (BSM) to assess the same hypothesis. This study aimed at determining the value of 128-lead BSM of P-waves in SR in predicting 12-month freedom from arrhythmia rate after initially successful DCCV for AF.

**Table 1:** Demographics, clinical outcomes, and medication details of study cohort.

Outcome at 12 months	Success (*n* = 23)	Failure (*n* = 29)	*p*-value
Demographics			
Age (years)	64 ± 5.6	63.6 ± 3.5	0.89
Acute success (%)	22 (100%)	28 (97%)	0.4
Immediate reversion to AF (%)	0 (0%)	6 (20%)	0.026
AF duration (months)	11.6 ± 3.2	13.5 ± 2.8	0.32
Required more than 1 shock (%)	2 (9%)	1 (3%)	0.39
Conscious sedation (%)	21 (95%)	29 (97%)	0.83
Previous DCCV (%)	5 (22%)	5 (17%)	0.53
DCCV within 12 months (%)	0 (0%)	4 (12%)	0.08
Ablation within 12 months (%)	0 (0%)	4 (12%)	0.08
Diabetes mellitus (%)	3 (14%)	2 (7%)	0.41
Heart failure (%)	4 (18%)	11 (37%)	0.15
Cerebrovascular event (%)	2 (9%)	2 (7%)	0.75
Ischemic heart disease (%)	1 (5%)	3 (10%)	0.48
Hypertension (%)	12 (46%)	17 (57%)	0.88
Indexed left atrial volume (ml/m^2^)	63.6 ± 1.9	73.7 ± 2.1	0.25
Body mass index (kg/m^2^)	33.6 ± 1.5	32.3 ± 1.4	0.6
Anti-arrhythmic drugs			
Amiodarone	9 (39%)	14 (48%)	0.58
Sotalol	9 (41%)	8 (27%)	0.29
Flecainide	1 (5%)	0 (0%)	0.25
Bisoprolol	5 (23%)	12 (40%)	0.2
Diltiazem, verapamil	1 (5%)	1 (3%)	0.83
Anti-arrhythmic drug not stopped (long term)	18 (82%)	22 (73%)	0.42

## Methods

This case–control study included 56 patients who underwent DCCV for AF between October 2013 and May 2015. DCCV acute success was defined as the immediate return to SR. Long-term DCCV success was determined by the lack of AF or atrial flutter up to 1 year following DCCV. P-wave parameters measured using BSM were used to predict DCCV outcome at 12 months and recurrence time. Clinical details of DCCV, follow-ups, and medication were obtained by retrospective analysis of clinical paper records. This study was a part of the USURP-AF II study approved by the East Midlands–Leicester Central Research Ethics Committee. REC reference: 19/EM/003

The selection criteria included the following:

1. Patients with persistent atrial fibrillation (persAF) who underwent external DCCV in Glenfield Hospital, Leicester, UK, between January 2013 and May 2015
2. Patients >18 years old and who consented to the study
3. Planting anterior leads around the breast tissue in females could be challenging. Therefore, only male patients were selected.
4. Patients who had ECG-documented AF directly before DCCV
5. Patients who were adequately anti-coagulated for 4 weeks before DCCV [therapeutic international normalised ratio (INR)].

**Figure 1:**
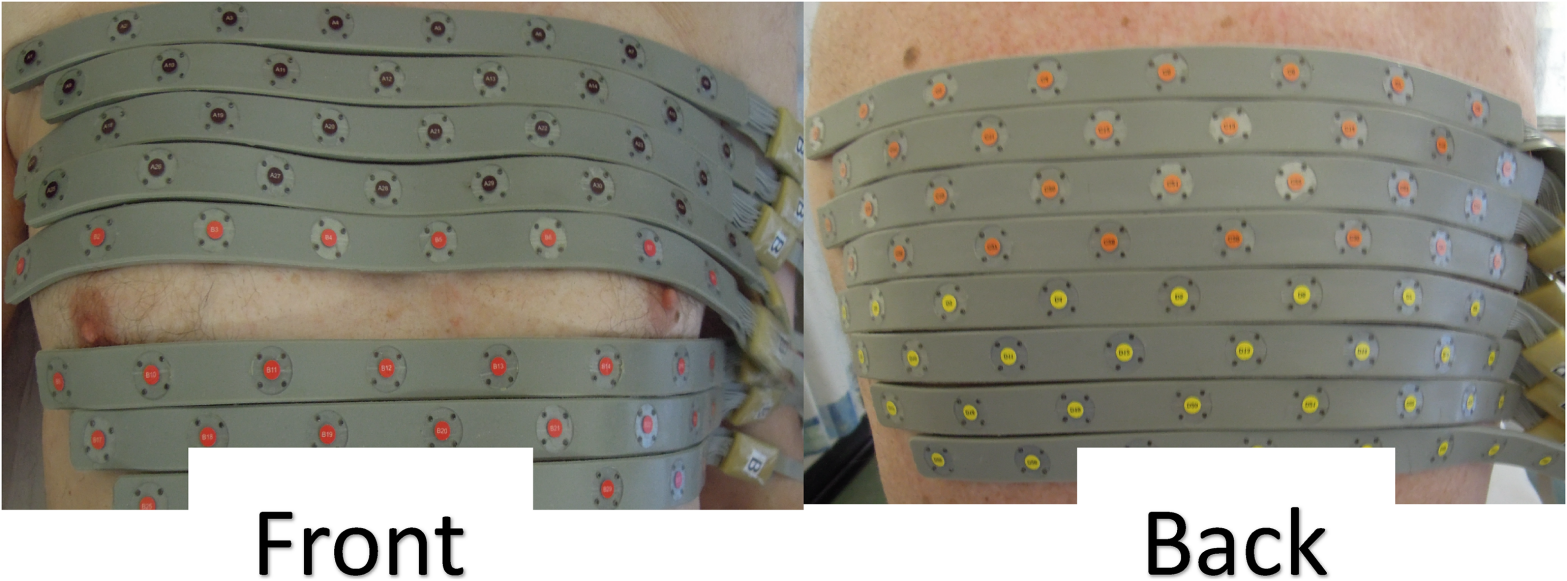
Demonstration of real-time body surface mapping.

P-wave parameter collection after DCCV for AF was done using 128 unipolar leads with 64 leads on the front and back of the torso (Figure 1). The vest utilised electrodes from Biosemi (Amsterdam, The Netherlands). The electrodes record digital ECGs before, during, and after DCCV. If DCCV was acutely successful, SR resumption would allow recording digital P-waves following DCCV. A MATLAB code was then applied to the digital BSM data using a band-pass filter of 1–50 Hz. It anointed the isoelectric line and P-wave peak to measure the P-wave duration (PWD) and amplitude. Also, it allowed the measurement of the P-wave manually by adjusting its start and end. The first four identifiable P-waves were measured and averaged in each lead. The parameters measured include the following:

The following P-wave parameters were produced:

1. PWD: Distance from P-wave onset to offset. It demonstrates a marker of atrial conduction.
2. P-wave amplitude (PWA): The area under the P-wave was estimated using the trapezoidal method by integrating the total area into a little trapezoid. It demonstrates atrial voltage. Regarding biphasic P-waves, the highest PWA absolute value between the positive and negative phases was accepted.
3. P-wave dispersion (PWDisp): The maximum difference between P-wave durations. It demonstrates atrial depolarisation heterogeneity.

**Figure 2:**
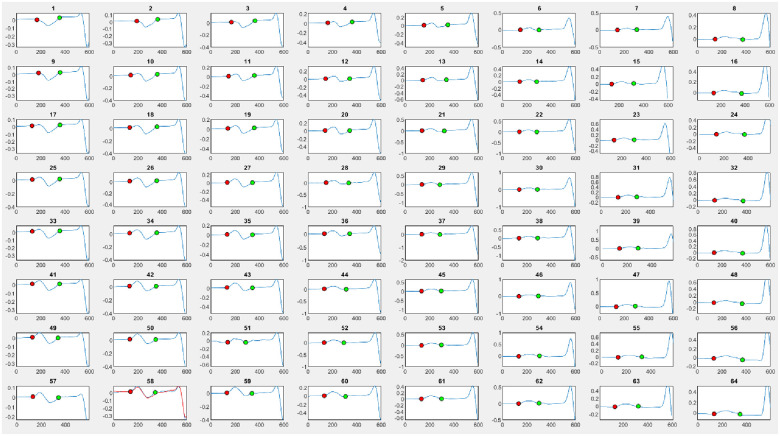
Demonstration of P-wave parameters anointing using body surface mapping at the front 64 leads. The resulting parameters are P-wave duration, amplitude, and dispersion. The red dot represents the beginning of the P-wave, while the green dot represents the end of the P-wave.

Each measurement was averaged in all 128 leads to produce one number representing a P-wave measurement in each patient (Figure 2).

In patients with immediate reversion to AF with <4 sinus beats, P-waves were measured until AF reversion. Heart rate (HR) was also noted in every ECG. Amiodarone is known to adjust P-wave morphology (14). Therefore, P-wave analysis was done after stratifying the patients based on amiodarone. It is well recognised that changes in HR and cycle length may affect P-wave morphology (15, 16). Previous studies have tried to tackle this by adjusting P-wave parameters to heart rate in regression analysis, a method that was not utilised in this study (17). As there are no current verified formulae to correct PWD for heart rate, we utilised a QT correcting formula to address this issue. Out of the formulae used in the literature, the Hodges formula has been proposed to be the most reliable and was therefore utilised in this study to correct PWD for heart rate, producing corrected P-wave duration (PWDc) (18). The intra-observer variability test anonymously analysed 20 BSMs on 2 days.

## Statistical analysis

Statistical analysis was conducted using GraphPad Prism V9.3 (San Diego, CA, USA). Categorical variables were expressed as frequency and percentage. The mean ± standard error of the mean was adopted to describe continuous parametric data. Unpaired t-tests or Mann–Whitney *U* tests were utilised to analyse unmatched data depending on the normality of the distribution. Gubbs’ test was conducted to identify outlier measurements. The cut-off of P-wave parameters for the DCCV outcome was analysed using receiver operator curve (ROC) analysis. The detected cut-off was used in a Kaplan–Meier survival analysis to use the proposed cut-off in predicting DCCV outcomes. *P*-value ≤0.05 represented statistical significance.

## Results

**Figure 3:**
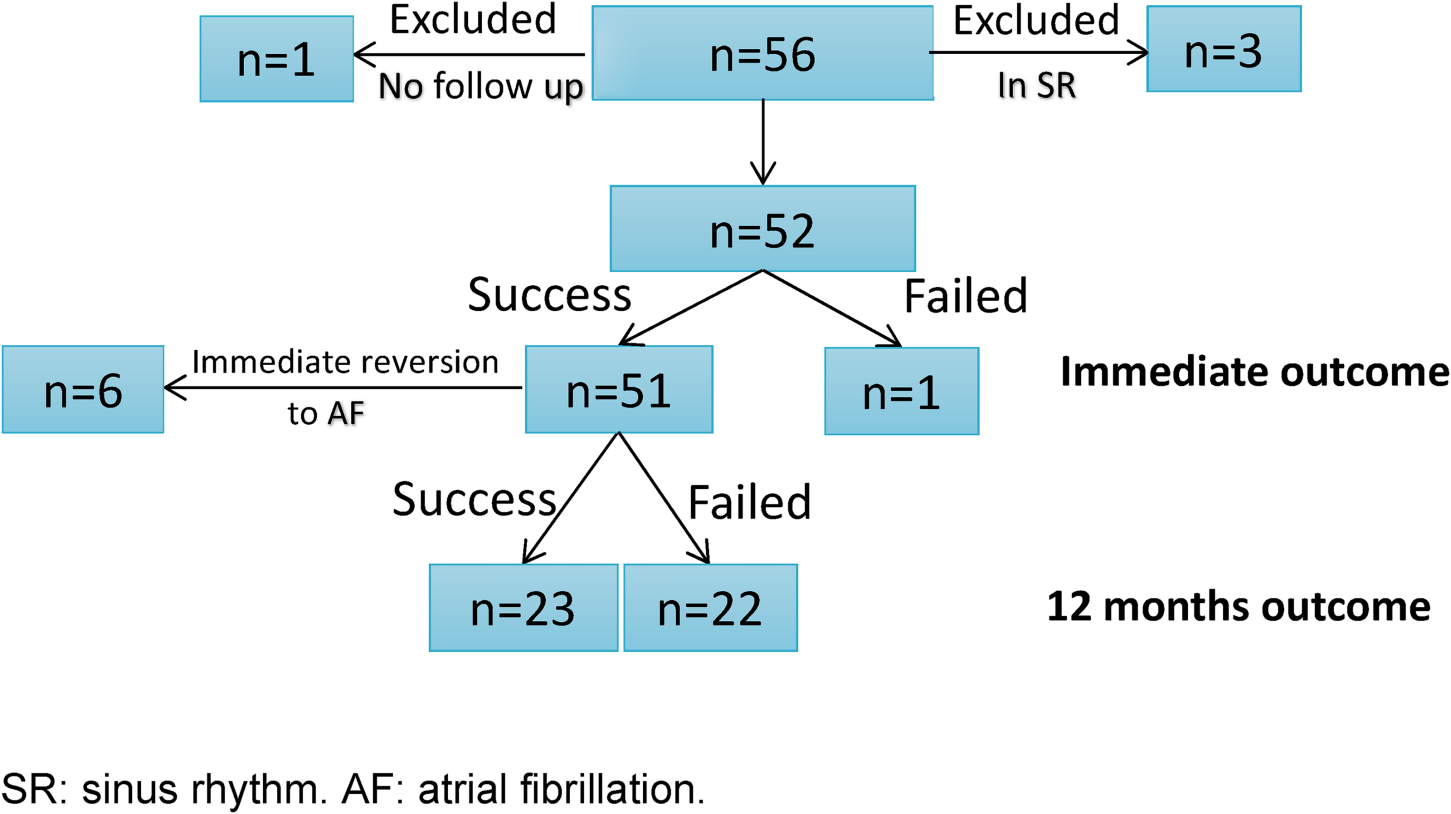
Clinical outcomes of the cohort.

The mean age was 64 ± 4 years. Three of the 56 patients involved were in SR at their DCCV appointment and were excluded. One patient was also excluded due to the lack of follow-up at 12 months (Figure 3). The 12-month success rate was 44% (*n* = 23), while the rest reverted to AF after 2.6 ± 0.4 months. Patients’ clinical details and demographics are provided in Table 2. Intra-observer variability was highest in PWA at 10% (0.05 mV), followed by PWDisp at 8% (9.5 ms) and PWD at 5% (8.4 ms). Gubbs’ test did not yield outlier values.

**Table 2:** Studies that correlated P-wave parameter with direct current cardioversion outcome.

Author and year	AF	*n*	Follow-up	ECG	Parameter	Recurrence change	Recurrence cut-off	*p*
Opolski et al., 1997 (19)	PersAF	35	6 months	SAECG	PWD	↑	>137 ms	<0.0001
Stafford et al., 1998 (20)	PersAF (77%)	31	1 week	SAECG	P-wave energy	↑	>25% drop	0.03
Aytemir et al., 1999 (21)	PersAF	73	6 months	SAECG	Filtered PWD	↑	>128 ms	0.001
Raitt et al., 2000 (22)	PersAF	20	1 year	SAECG	PWD	↑	>130–140 ms	0.005
Guo et al., 2003 (23)	PersAF	60	6 months	SAECG	Filtered PWD	↑	Nil	<0.0001
Ehrlich et al., 2003 (24)	No mention	111	1 week	SAECG	PWD	↑	>145 ms	<0.001
Dixen et al., 2004 (25)	PersAF	131	1 month	SAECG	PWD	↑	>160 ms	0.03
Dogan et al., 2004 (26)	PersAF (45%)	64	6 months	SAECG	PWDisp	↑	>46 ms	<0.001
Perzanowski et al., 2005 (27)	PersAF	45	6 months	SAECG	PWDisp	↑	>80 ms	0.05
Budeus et al., 2005 (28)	PersAF	141	1 year	SAECG	PWD	↑	>126 ms	0.0001
Başar et al., 2011 (29)	PersAF	26	1 year	12 leads	PWDisp	↑		0.001
Gonna et al., 2014 (30)	PersAF	77	1 month	12 leads	PWD	↑	>125 ms	0.025
Blanche et al., 2014 (31)	PersAF	133	9 months	SAECG	Nil	Nil	Nil	Non-significant
Fujimoto et al., 2018 (32)	PersAF	141	1 month	12 leads	PWDisp	↑	Nil	0.001
Choi et al., 2021 (12)	PersAF	272	2 months	12 leads	PWD PTFV1	↑	>134 ms >50 mm.ms	0.012 0.011

PTFV1, P-wave terminal force in V1.

P-wave parameters were compared between successful and failed arms (PWDc: 163 vs. 178 ms, *P* = 0.29. PWA: 0.33 vs. 0.38 mV, *P* = 0.1. PWDisp: 24 vs. 27 ms, *P* = 0.35).

**Figure 4:**
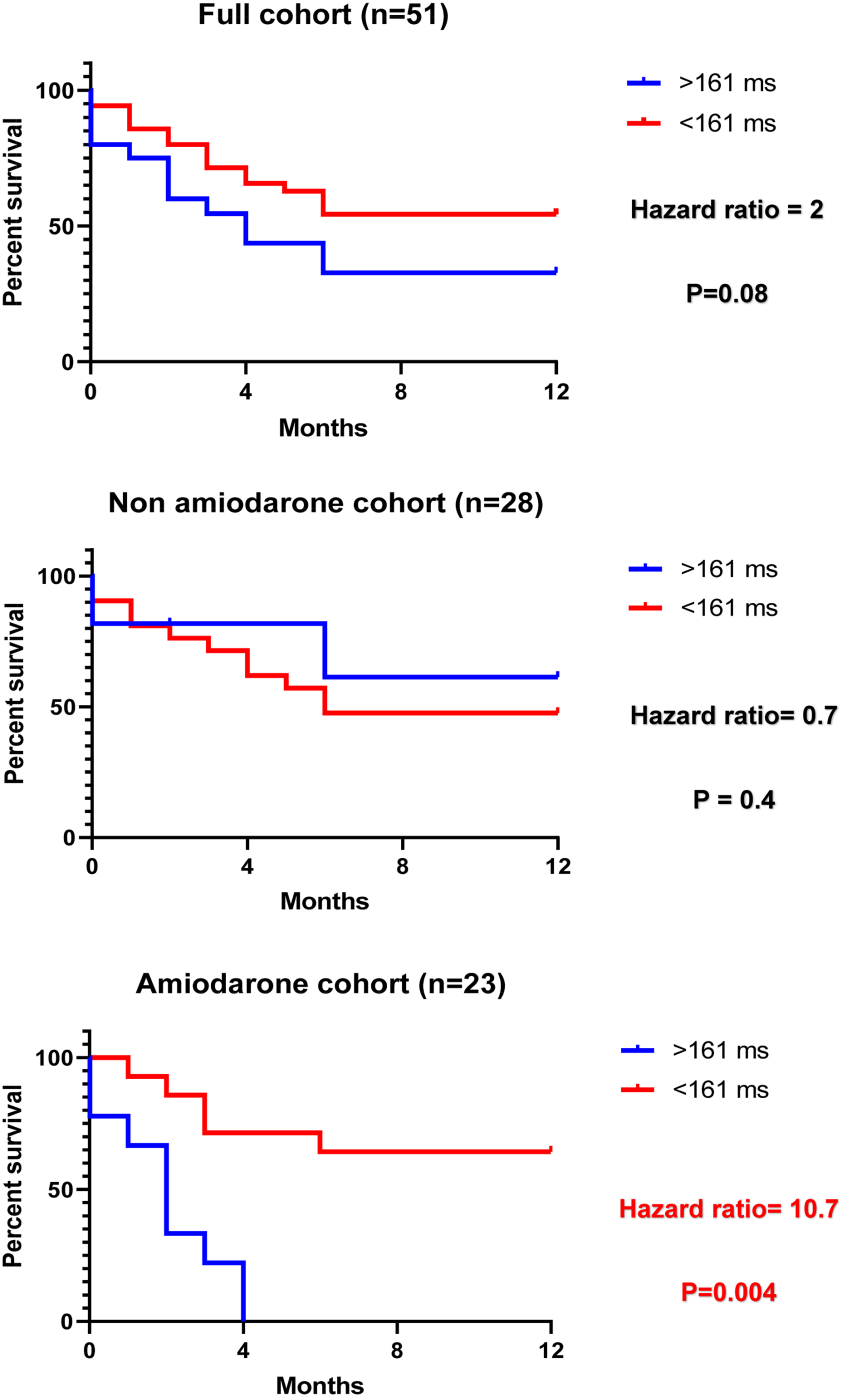
Kaplan–Meier survival analysis for the corrected P-wave duration cut-off in all cohorts.

After stratifying for amiodarone, the success rates in the amiodarone and non-amiodarone cohorts were 39% vs. 48%, *P* = 0.58, respectively. P-wave parameters were comparable in the non-amiodarone cohort (172 vs. 167 ms, *P* = 0.82; PWA: 0.34 vs. 0.41 mV, *P* = 0.2; PWDisp: 26 vs. 24 ms, *P* = 0.58). In patients on amiodarone, those who had failed arms had higher PWDc than those with successful arms (188 vs. 150 ms, *P* = 0.04), while PWA and PWDisp were comparable between both arms (0.3 vs. 0.4 mV, *P* = 0.09, and 21 vs. 20 ms, *P* = 0.94, respectively). ROC curve analysis for PWDc in the amiodarone cohort showed an area under the curve (AUC) of 0.75 and *P* = 0.049. A recurrence cut-off >161 ms had a sensitivity of 69% and a specificity of 100%. The Kaplan–Meier survival analysis using a PWDc cut-off of 161 ms in the amiodarone cohort indicated a hazard ratio of 10.7, *P* = 0.004. By contrast, the non-amiodarone and full cohorts did not show a difference between cut-off and 12-month DCCV outcomes (Figure 4). There was no correlation between P-wave parameters and AF recurrence time in the entire cohort (PWDc: *r* = −0.23, *P* = 0.24; PWA: *r* = 0.33, *P* = 0.09; PWDisp: *r* = −0.13, *P* = 0.52), the amiodarone cohort (PWDc: *r* = −0.02, *P* = 0.95; PWA: *r* = 0.5, *P* = 0.07; PWDisp: *r* = –0.23, *P* = 0.45), and the non-amiodarone cohort (PWDc: *r* = −0.36, *P* = 0.24; PWA: *r* = −0.1, *P* = 0.74; PWDisp: *r* = −0.12, *P* = 0.71).

## Discussion

Although other studies correlated P-wave parameters after DCCV with outcome using signal-averaged ECG or 12-lead ECG (summarised in Table 2), this is the first study to perform this using BSM of P-waves. The 1-year success rate in our cohort falls into the higher range of previous studies demonstrating relatively good outcomes in our centre. However, the inclusion of males might have positively affected the one-year success rate (33). Unlike former studies, demographics in this cohort were not predictive of the outcome (34, 35). One would expect a positive correlation of AF risk factors with AF recurrence after DCCV. It was noted that this cohort only involved male patients. This could cause selection bias and affect the rest of the demographics. Therefore, further studies with a random selection of subjects would be advised to investigate predictive factors (36, 37).

Regarding P-wave analysis, to the best of our knowledge, no previous study has correlated P-wave BSM with 12 months of DCCV outcome and assessed recurrence time. Stratification by being on amiodarone was conducted to address its effects on P-wave parameters. Increased *PWDc* after DCCV in the amiodarone cohort was associated with 12-month failure with high specificity. This was in keeping with previous studies in Table 2. This can be explained by left atrium (LA) remodelling, fibrosis, and intra-atrial conduction delay in patients prone to AF recurrence (38). Furthermore, a previous study demonstrated shorter PWD in patients with AF successfully treated with amiodarone (14). These results were not evident in patients who were not on amiodarone. The reason behind this finding is unclear. One reason could be that patients did not have refractory AF significant enough to be on amiodarone, leading to the lack of LA conduction delay. This may be possible to detect with imaging modalities and is advised in future studies. Another reason could be the potential small size to detect a significant effect. As for *PWA*, one would expect a correlation between low PWA (caused by fibrosis) and DCCV 1-year failure. This corresponded to the PVI studies that proposed this (39, 40). Although only one study correlated reduced PWA with AF recurrence after internal cardioversion (17), DCCV (external) was used in this study, and all studies in Table 2 did not show similar findings. It was unclear why PWA was predictive of outcome in internal DCCV but not external DCCV. However, one theory is that external cardioversion could have caused lower atrial stunning, not affecting PWA, unlike internal cardioversion (41). Further randomised trials would be beneficial in establishing a mechanism.

*PWDisp* reflects inhomogeneous atrial refractoriness and AF vulnerability (42). It was not correlated to the DCCV 12-month outcome in this cohort. According to previous studies, amiodarone decreases PWDisp because of increased atrial repolarisation (14, 43). Following the hypothesis that the amiodarone cohort had more LA remodelling, it is possible that the increased PWDisp from remodelling was decreased to normal by the amiodarone effect. Patients not on amiodarone did not have enough remodelling to cause a notable PWDisp effect predictive of DCCV outcomes per previous studies (Table 2). These results warrant further investigation into predicting DCCV outcomes in patients on and off amiodarone. Further studies should directly compare BSM, signal-averaged electrocardiogram (SAECG), and 12-lead ECG in utilising P-wave parameters to predict 12-month DCCV outcome for AF.

## Conclusion

Predicting DCCV outcome using P-wave parameters measured in SR using BSM was only feasible in patients on amiodarone. In this cohort, increased PWDc in SR directly following DCCV was associated with failed DCCV at 12 months. PWDc >161 ms was 100% specific for AF recurrence by 12 months after initial successful DCCV. This could serve as a marker for considering an early rate control strategy.

## Limitations

This is a single-centre retrospective study with AF recurrence detected using 12-lead ECG or Holter monitoring. Long-term monitoring was not done, and the AF burden was not evaluated. This could have missed sub-clinical and micro-AF episodes. The relatively low sample size with post-hoc power analysis of 67% and 71% in the full cohort and amiodarone cohort to detect a significant difference in PWDc may lead to a type-2 error. Therefore, future studies using BSM with a higher number and pre-study sample size calculation are advised. Direct comparison between BSM, 12-lead ECG, and SAECG was not conducted and is suggested in future studies. This study only included male patients, which can cause selection bias. Although not impossible, including female patients would require the adjustment of lead positions, making comparisons challenging. Flecainide and sotalol used in our cohort affected PWD (44, 45). Furthermore, patients stopping their anti-arrhythmic drugs were included in the analysis. Future studies matching patients by anti-arrhythmic drugs and their cessation are needed to limit confounding. The Hodges formula is currently not verified as a methodology in the literature to correct PWD for HR. A future dedicated study would be useful to confirm the utility of this formula for the benefit of future studies utilising PWD. One of the main limitations of this study is the age of the data utilised. The data were collected between 2013 and 2015, which may affect the applicability of the findings to current clinical practice. In future studies, incorporating recent data with longer follow-ups is advised. This study did not measure potential relevant pre-DCCV parameters, including AF cycle length and AF coarseness. The study did not utilise magnetic resonance imaging to evaluate LA fibrosis, which is recommended for future studies.

## Data Availability

The raw data supporting the conclusions of this article will be made available by the authors without undue reservation.
